# SOCS2 Downregulation Is Associated with a Ferro-Aging-Related Transcriptomic Signature in Proximal Tubular Cells in Chronic Kidney Disease

**DOI:** 10.3390/biomedicines14092047

**Published:** 2026-09-11

**Authors:** Bin Xia, Rujie Zhou, Jianglong Chen, Guang Li

**Affiliations:** Yunnan Branch of Institute of Medicinal Plant Development, Chinese Academy of Medical Sciences & Peking Union Medical College, Jinghong 666100, China

**Keywords:** ferroptosis, chronic kidney disease, biomarker discovery, proximal tubular

## Abstract

**Objectives:** This study aimed to identify ferro-aging-related hub genes in chronic kidney disease (CKD) and elucidate their potential roles in proximal tubular (PT) cells. **Methods:** Differential expression analysis was performed using GSE104954, and the differentially expressed genes were intersected with a set of ferro-aging-related genes. Feature selection was conducted using the least absolute shrinkage and selection operator (LASSO), support vector machine–recursive feature elimination (SVM-RFE), and random-forest algorithms. The findings were externally validated in GSE180394. Single-cell transcriptomic data from GSE183276 were then used to define the cellular localization and expression profile of the hub genes, and their regulatory networks were investigated using scTenifoldKnk-based in silico knockout and gene set enrichment analysis (GSEA). **Results:** Fourteen candidate genes were identified, of which IRF7 and SOCS2 were consistently selected by all three machine-learning algorithms. SOCS2 was significantly downregulated in both the discovery and validation cohorts and showed strong tissue-level discriminatory performance in the discovery cohort, with more modest performance in the external cohort. Single-cell analysis showed that SOCS2 downregulation occurred predominantly in PT cells; CKD PT cells also exhibited increased ACSL4 expression and the enrichment of the ferroptosis pathway. In silico SOCS2 knockout further identified 77 significantly perturbed genes, primarily associated with mitochondrial oxidative phosphorylation, oxidative stress, and metabolic homeostasis. **Conclusions:** Collectively, these findings identify SOCS2 as a candidate gene associated with a ferro-aging-related transcriptomic signature in CKD PT cells. The observed changes in ACSL4, ferroptosis-related pathways, mitochondrial oxidative phosphorylation, and oxidative stress networks are compatible with, but do not establish, a ferro-aging phenotype. Further functional validation is required to determine whether SOCS2 contributes causally to iron–lipid dysregulation and tubular injury in CKD.

## 1. Introduction

Chronic kidney disease (CKD) is a progressive disorder characterized by persistent abnormalities in kidney structure and function [1]. The global burden of CKD continues to increase with population aging and the rising prevalence of metabolic disorders such as diabetes and hypertension [2]. Because the early clinical manifestations of CKD are often insidious, some patients already have irreversible kidney damage at the time of diagnosis. Elucidating the molecular mechanisms underlying CKD onset and progression is therefore essential for developing effective interventions [1].

Tubulointerstitial injury is a major pathological driver of CKD progression. Proximal tubular (PT) cells, which mediate reabsorption, energy metabolism, and the transport of toxic metabolites, are particularly susceptible to hypoxia, inflammation, oxidative stress, and uremic toxins [3,4]. Recent studies indicate that PT cells are not merely passive targets of injury; they also reshape the renal inflammatory microenvironment by releasing cytokines, chemokines, and damage-associated signals [5]. Persistent PT-cell injury can further induce mitochondrial dysfunction, impaired oxidative phosphorylation, and reactive oxygen species (ROS) accumulation, thereby promoting tubular atrophy, interstitial inflammation, and fibrosis [4,5]. PT cells may therefore act as key effector cells that amplify renal injury during CKD.

Disrupted iron homeostasis increases the labile intracellular iron pool and promotes reactive oxygen species (ROS) generation via the Fenton reaction, thereby enhancing lipid peroxidation [5,6]. Ferroptosis is a form of regulated cell death driven by iron-dependent phospholipid peroxidation that ultimately results in lethal membrane damage and the loss of cellular viability [6,7]. In particular, ACSL4 promotes the incorporation of polyunsaturated fatty acids into membrane phospholipids and is a key determinant of cellular susceptibility to ferroptosis [8]. In contrast, the recently proposed concept of ferro-aging describes an age-progressive and chronic iron–lipid dysregulation state in which persistent iron accumulation and lipid peroxidation promote oxidative stress, mitochondrial dysfunction, cellular senescence, and progressive functional decline without necessarily causing immediate cell death [9,10]. Thus, although ferro-aging and ferroptosis share several molecular features, including iron dyshomeostasis, ROS generation, and lipid peroxidation, they differ principally in temporal scale and biological outcome: ferroptosis represents a regulated cell death endpoint, whereas ferro-aging refers to a chronic aging-associated trajectory that may persist under sublethal oxidative stress and potentially increase susceptibility to subsequent ferroptotic injury [9,10,11,12]. Senescent cells can accumulate substantial amounts of intracellular iron while remaining resistant to ferroptosis, further indicating that chronic iron accumulation and cellular aging do not necessarily equate to ferroptotic cell death [11].

In CKD, persistent inflammation, uremic toxin exposure, mitochondrial dysfunction, and impaired redox homeostasis may provide a biological context in which chronic iron and lipid-peroxidation abnormalities accumulate in proximal tubular cells [4,5,7]. The upregulation of ACSL4, downregulation of GPX4, and dysregulation of genes involved in iron transport and storage may therefore represent molecular features compatible with a ferro-aging-like state [7,8,9]. However, ferro-aging remains an emerging concept, and its presence cannot be inferred solely from transcriptomic alterations. Accordingly, in the present study, ferro-aging is used as a hypothesis-generating framework for identifying CKD-associated molecular signatures rather than as a directly demonstrated cellular phenotype.

Accordingly, this study integrated bulk transcriptomics, single-cell transcriptomics, machine learning, and in silico knockout analysis to identify ferro-aging-related hub genes in CKD, characterize the expression and cellular localization of SOCS2 in the kidney, and investigate its potential regulatory role in ferro-aging-related PT-cell injury. Specifically, we asked whether SOCS2 is consistently associated with CKD-related proximal tubular injury and whether its expression and predicted regulatory network are linked to molecular features compatible with a ferro-aging-like state.

## 2. Materials and Methods

### 2.1. Data Sources

GSE104954 [13] was used as the discovery cohort and included 169 CKD samples and 21 living-donor controls from microdissected tubulointerstitial kidney tissue profiled on two microarray platforms (GPL22945 and GPL24120). The CKD samples represented multiple renal disease categories. Five tumor-nephrectomy samples were excluded from the present analysis.

GSE180394 [14] served as the external validation cohort and included 44 renal disease samples and 15 controls from microdissected tubular kidney tissue profiled on GPL19983. The controls comprised nine living-donor samples and six non-neoplastic kidney samples from tumor nephrectomy.

GSE183276 [15] was used for single-cell analysis. After exclusion of AKI donors, the CKD/control analysis included 33 unique donors represented by 37 libraries and 45,112 cells, comprising 15 CKD donors and 18 healthy reference donors. Detailed cohort characteristics, renal diagnoses, control sources, and inclusion/exclusion criteria are summarized in Table 1.

**Figure 1:**
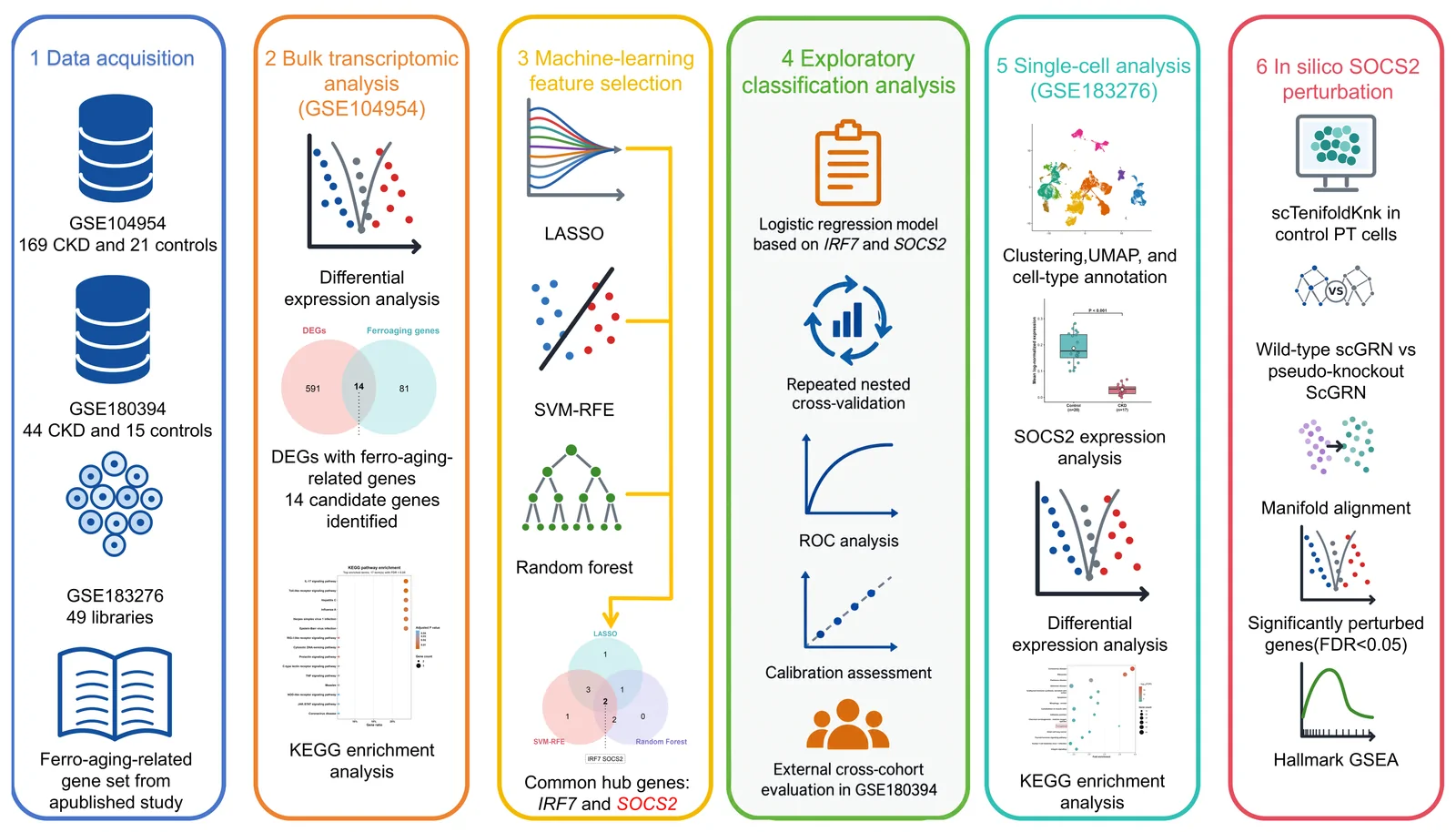
Overall study design and analytical workflow. The study integrated bulk transcriptomic analysis, machine-learning feature selection, external validation, single-cell transcriptomic analysis, and in silico SOCS2 perturbation. GSE104954 was used as the training cohort for differential expression analysis and candidate-gene identification; GSE180394 was used for external validation; and GSE183276 was used for the single-cell characterization of SOCS2 expression and proximal tubular cell transcriptomic alterations. SOCS2-associated regulatory perturbations were further explored using scTenifoldKnk (scTenifoldKnk 1.0.3) and Hallmark GSEA.

The ferro-aging-related gene set was obtained from a published study [9]. The ferro-aging-related gene set comprised 95 genes obtained from the study by Liu et al. [9], which introduced ferro-aging as an aging-associated iron–lipid dysregulation axis characterized by age-progressive iron accumulation, chronic lipid peroxidation, ACSL4 activation, and cellular senescence across human and non-human primate tissues. The complete ferro-aging-related gene list used in the present analysis is provided in Supplementary Table S1. Because this gene set was originally defined in an aging context rather than specifically in CKD kidney tissue, its application to CKD was considered exploratory and hypothesis-generating. The overall study design and analytical workflow are summarized in Figure 1.

### 2.2. Differential Expression Analysis of GSE104954

For GSE104954, the GPL22945 and GPL24120 expression matrices were processed separately before cross-platform integration. Expression distributions were inspected to determine whether log2 transformation was required; values from both platforms were already on a log2 scale and were therefore not transformed again. Probes without valid gene-symbol annotations were removed, and multiple probes corresponding to the same gene symbol were collapsed by calculating their mean expression. No missing expression values were detected in either platform. A total of 12,042 gene symbols shared by both platforms were retained, and the two matrices were merged by gene symbol and quantile-normalized using the normalizeBetweenArrays function in limma (limma 3.68.4) [16]. Reporting of the microarray preprocessing workflow was guided by established MIAME principles [17].

For differential expression analysis, the quantile-normalized matrix was analyzed using a limma design matrix containing both disease group and platform, and the CKD-versus-control contrast was estimated while adjusting for platform. For visualization and subsequent exploratory feature analyses, platform effects were additionally removed using limma::removeBatchEffect, with the CKD–control design retained to avoid removing the biological group contrast. PCA was performed before and after platform-effect correction to evaluate changes in platform-related separation.

### 2.3. Identification of Ferro-Aging–Related Candidate Genes

Significantly differentially expressed genes in GSE104954 were intersected with the ferro-aging-related gene set to identify candidate genes.

### 2.4. KEGG Enrichment Analysis of Candidate Genes

Gene symbols were converted to ENTREZ identifiers using AnnotationDbi (AnnotationDbi 1.74.0) and org.Hs.eg.db (org.Hs.eg.db 3.23.1). Kyoto Encyclopedia of Genes and Genomes (KEGG) enrichment analysis of the 14 candidate genes was performed using the 11,996 successfully annotated genes in GSE104954 as the background. KEGG annotations were retrieved using KEGGREST (KEGGREST 1.52.2), and only canonical human pathways were retained. Gene set sizes were restricted to 10–500 genes. A one-sided hypergeometric test was used, and *p* values were adjusted using the Benjamini–Hochberg method. An FDR < 0.05 was considered statistically significant.

### 2.5. Machine-Learning Identification of Candidate Hub Genes

The 14 candidate genes were used as features for diagnostic-gene selection with LASSO, SVM-RFE, and random-forest algorithms. For LASSO, a binomial logistic regression model was fitted using glmnet (glmnet 5.0) [18], and genes with nonzero coefficients were selected by stratified 10-fold cross-validation. SVM-RFE [19] was implemented with a linear-kernel support vector machine and evaluated using balanced accuracy. The random forest [20] comprised 1000 decision trees and used balanced bootstrap sampling to address class imbalance. Genes selected by all three algorithms were designated as candidate hub genes for subsequent exploratory analyses.

To obtain an optimism-corrected estimate of the complete model-development workflow, we performed repeated nested cross-validation [21]. The outer loop consisted of stratified fivefold cross-validation repeated five times, with stratification by disease status and microarray platform. Within each outer training set, platform effects were estimated using gene-wise linear models that retained the CKD indicator, and the estimated platform coefficients were applied to the corresponding training and held-out samples using platform identity only. Differential expression filtering, intersection with the prespecified ferro-aging gene set, LASSO, SVM-RFE, random-forest selection, the three-method intersection, logistic-model fitting, and cutoff selection were all repeated within the outer training set. LASSO and SVM-RFE tuning used stratified fivefold inner cross-validation, whereas random-forest tuning used balanced bootstrap sampling and out-of-bag balanced accuracy. The Youden cutoff was derived from each outer training set and then applied to its held-out fold. Subject-level probabilities were averaged across the five repeated outer-test predictions, and classifications were summarized by majority vote. Performance estimates and 95% confidence intervals were calculated using 2000 stratified bootstrap resamples.

### 2.6. Logistic Regression Model and Internal Evaluation

A multivariable logistic regression model was constructed using disease status as the dependent variable and IRF7 and SOCS2 expression as independent variables:

logit(P)=−5.808765+7.745723×IRF7−5.198669×SOCS2

No additional z-score standardization or min–max scaling was applied to IRF7 or SOCS2 before logistic regression. The model used preprocessed expression values that had been confirmed to be on the log2 scale, quantile-normalized, and corrected for platform effects, rather than raw expression intensities.

The primary internal performance assessment was based on the out-of-fold predictions from the repeated nested cross-validation described above. Discrimination was summarized using the AUC, sensitivity, specificity, balanced accuracy, accuracy, positive predictive value, and negative predictive value. Calibration was assessed using the Brier score, calibration intercept, calibration slope, and a grouped calibration plot. Confidence intervals were obtained from 2000 stratified bootstrap resamples of the subject-level out-of-fold predictions. After internal validation, the complete development procedure was refitted in the full GSE104954 cohort to obtain the final model. This full-cohort analysis recovered IRF7 and SOCS2 and reproduced the equation shown above. The discovery-cohort Youden cutoff of 0.899343941 was locked before external application.

### 2.7. External Validation

External validation was performed in GSE180394 by applying the original GSE104954 IRF7/SOCS2 equation without coefficient refitting. The discovery-cohort cutoff of 0.899343941 was specified before examining external performance and was applied unchanged; no cutoff was selected using GSE180394 outcomes. Because GPL19983 was an unseen platform, no disease-informed external batch adjustment or outcome-dependent recalibration was performed. Discrimination was evaluated using the AUC, and threshold-based performance was summarized using sensitivity, specificity, balanced accuracy, accuracy, positive predictive value, and negative predictive value. Calibration was evaluated using the Brier score, calibration intercept, calibration slope, and a grouped calibration plot. Ninety-five percent confidence intervals were obtained from 2000 stratified bootstrap resamples. These analyses were intended to assess the cross-cohort reproducibility of tissue-expression discrimination rather than clinical diagnostic utility [22].

### 2.8. Single-Cell Data Processing and Cell-Type Annotation

GSE183276 contained 37,080 genes and 109,741 cells from 49 libraries. Quality control was performed separately for each library. Low-quality cells were filtered using the median absolute deviation (MAD) method, with thresholds of nCount_RNA ≥ 300, nFeature_RNA ≥ 200, and mitochondrial and endoplasmic-reticulum gene proportions ≤ 40%, initially retaining 81,681 cells. Ambient RNA contamination was then corrected using decontX (celda 1.28.0), and doublets were removed using scDblFinder (scDblFinder 1.26.2), yielding 68,154 high-quality cells. After acute kidney injury samples were excluded, 31,257 cells from the CKD group and 13,855 cells from the control group were included. Log normalization was performed using scater [23], and 3000 highly variable genes were selected using scran (scran 1.40.0) [24]. A shared nearest-neighbor graph was constructed from the first 50 principal components, followed by Leiden clustering, which identified 26 clusters at a resolution of 2.0. Uniform manifold approximation and projection (UMAP) was used for dimensionality-reduction visualization. Cell types were annotated using an integrated score based on canonical marker genes, average expression, the proportion of marker-positive cells, and z scores, followed by manual review of marker-expression plots. Multiple libraries derived from the same donor were retained during cell-level preprocessing but were not treated as independent biological replicates in donor-level statistical analyses.

### 2.9. SOCS2 Expression in PT Cells and Pseudobulk Differential Expression Analysis

After PT cells were extracted, SOCS2 expression analyses were performed at the donor level. For donors represented by multiple libraries, PT-cell expression summaries were aggregated across libraries so that each unique donor contributed only one biological observation to between-group statistical testing. Mean SOCS2 log1p(CP10K) expression and the proportion of SOCS2-positive cells were calculated for each donor, and differences between CKD and control donors were assessed using the Wilcoxon rank-sum test. Effect sizes were additionally quantified using Cohen’s d and Hedges’ g. Raw counts were aggregated at the donor-by-cell-type level to construct donor-level pseudobulk matrices. For donors represented by multiple libraries, counts were summed across libraries before downstream differential expression analysis. Differential expression analysis was performed using edgeR v4.10.1 [25], including low-expression filtering with filterByExpr, trimmed mean of M-values normalization, robust dispersion estimation, and quasi-likelihood generalized linear model testing. *p* values were adjusted using the Benjamini–Hochberg method, and genes with an adjusted *p* value < 0.05 and |log2FC| > 0.25 were considered significantly differentially expressed. The PT-cell analysis included 20 control and 17 CKD libraries, with 8273 genes tested.

### 2.10. KEGG Enrichment Analysis of PT-Cell Differentially Expressed Genes

The 1227 significantly differentially expressed genes in PT cells were used as input for KEGG enrichment analysis with the enricher function in clusterProfiler v4.20.0. Gene set sizes were restricted to 10–500 genes. A one-sided hypergeometric test was applied, and *p* values were adjusted using the Benjamini–Hochberg method. The primary analysis used the complete set of KEGG-annotated genes as the background, and a sensitivity analysis used the 8273 genes detected in the PT-cell pseudobulk differential expression analysis as the background. The KEGG ferroptosis pathway (hsa04216) was extracted separately, and pathview was used to visualize the differentially expressed genes in the pathway and their log2FC values.

### 2.11. In Silico SOCS2 Network Perturbation Analysis

To investigate the predicted regulatory consequences of SOCS2 perturbation in PT cells, control PT cells from the initial cell-type annotation were extracted, and the raw gene-by-cell expression matrix was used as input for scTenifoldKnk [26]. Briefly, scTenifoldKnk first constructs a wild-type single-cell gene regulatory network (scGRN) from the observed expression matrix. A pseudo-knockout network is then generated by copying the wild-type adjacency matrix and setting the entire row corresponding to the target gene, SOCS2, to zero, thereby removing its regulatory contribution from the inferred network. Thus, this procedure represents a network-level virtual gene deletion rather than complete suppression of SOCS2 expression in the original expression matrix or functional inactivation of the SOCS2 protein.

The wild-type and pseudo-knockout scGRNs were subsequently compared using manifold alignment. For each gene, a differential-regulation distance was calculated from the difference between its projected positions in the two aligned networks, with a larger distance indicating a greater predicted regulatory perturbation following virtual SOCS2 deletion. Statistical significance was assessed using the χ^2^-based procedure implemented in scTenifoldKnk, with one degree of freedom, followed by Benjamini–Hochberg correction for multiple testing. Genes with an FDR < 0.05 were defined as significantly perturbed genes. Because the differential-regulation distance reflects the magnitude of network perturbation rather than the direction of gene expression change, Hallmark GSEA was additionally performed using genes ranked by differential expression between CKD and control PT cells, and significantly enriched pathways were compared to the SOCS2-perturbed regulatory network.

### 2.12. Disease-Subtype Sensitivity Analyses of SOCS2 Expression

To assess whether SOCS2 downregulation was driven by specific renal disease categories rather than representing a broader CKD-associated pattern, disease-subtype sensitivity analyses were performed separately within each platform. Because disease composition and control distribution were strongly confounded with platform in GSE104954, disease subgroups from GPL22945 and GPL24120 were not directly pooled for inferential comparisons. Each disease subgroup was compared to the living-donor controls from the same platform. Effect sizes were quantified using Cohen’s d, and *p* values were adjusted using the Benjamini–Hochberg method.

In GSE180394, SOCS2 expression was additionally examined across five prespecified renal disease categories: lupus nephritis, FSGS/FGGS, diabetic nephropathy, IgA nephropathy, and other renal diseases. In GSE183276, donor-level PT-cell SOCS2 expression was analyzed separately in DKD and hypertensive CKD. Because several disease subgroups had small sample sizes, these analyses were considered sensitivity analyses and were interpreted primarily with respect to direction and effect size rather than statistical significance alone.

## 3. Results

### 3.1. Identification and Functional Characterization of Ferro-Aging-Related Candidate Genes in CKD

**Figure 2:**
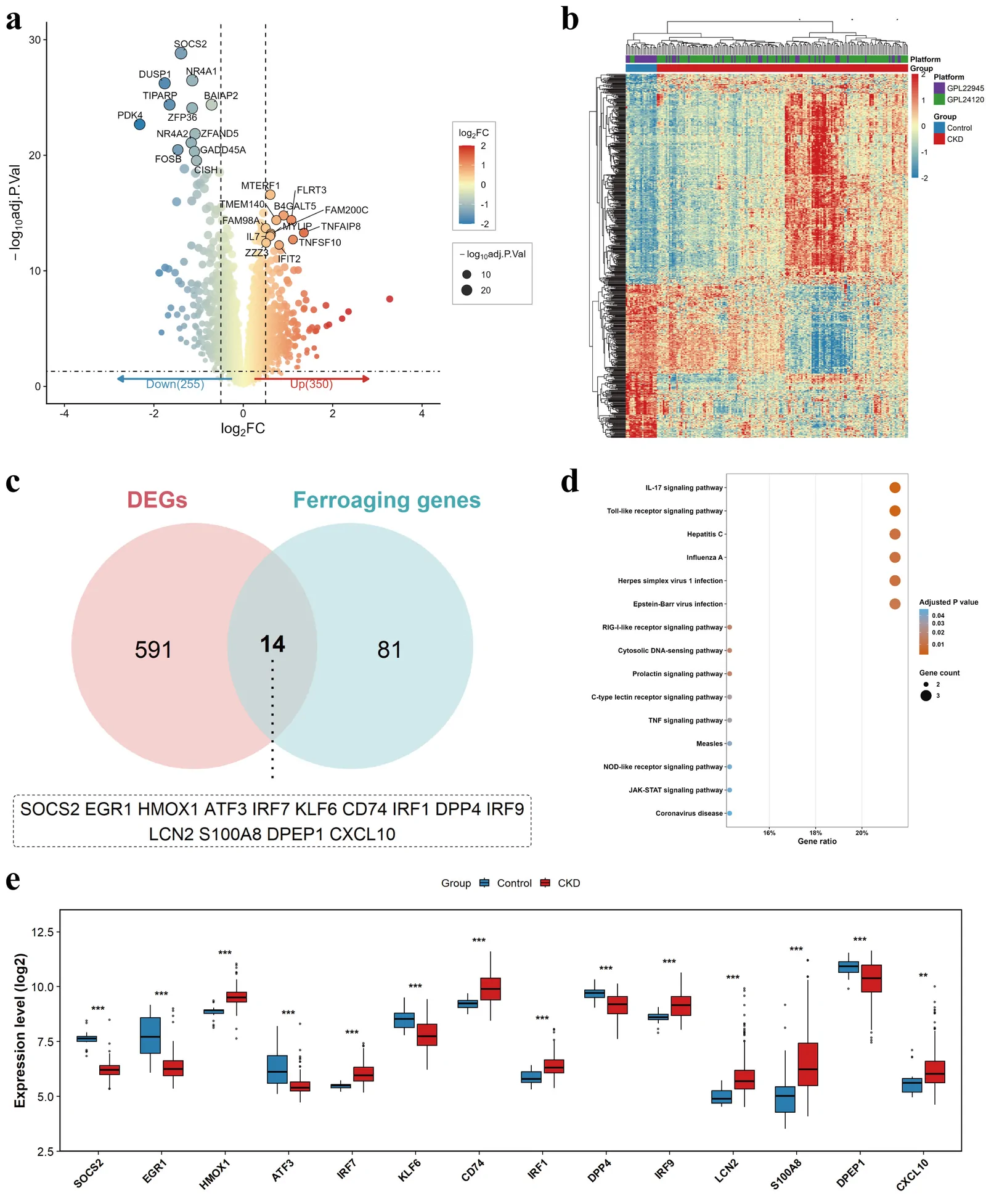
Identification of CKD-associated ferro-aging-related candidate genes in GSE104954. (**a**) Volcano plot of differential expression between CKD and control samples (FDR < 0.05 and |log2FC| > 0.5); (**b**) hierarchical clustering heatmap of differentially expressed genes; (**c**) intersection of differentially expressed genes with ferro-aging-related genes; (**d**) KEGG enrichment analysis of the intersecting genes; (**e**) expression of the intersecting genes in the two groups. Boxplots show the median and interquartile range, with whiskers extending to 1.5× the interquartile range. Between-group comparisons were performed using two-sided Wilcoxon rank-sum tests. ** *p* < 0.01; *** *p* < 0.001.

PCA before and after platform-effect correction is shown in Supplementary Figure S1. Differential expression analysis was performed after merging and correcting the data from the two microarray platforms in GSE104954. The volcano plot identified 605 differentially expressed genes between the CKD and control groups, including 350 upregulated and 255 downregulated genes (Figure 2a). Hierarchical clustering showed that the expression patterns of these genes clearly distinguished CKD from control samples (Figure 2b). Intersecting the differentially expressed genes with the ferro-aging gene set yielded 14 candidate genes: SOCS2, EGR1, HMOX1, ATF3, IRF7, KLF6, CD74, IRF1, DPP4, IRF9, LCN2, S100A8, DPEP1, and CXCL10 (Figure 2c). KEGG analysis showed that these genes were primarily enriched in inflammation- and stress-related pathways, including the IL-17, Toll-like receptor, TNF, NOD-like receptor, and JAK–STAT signaling pathways (Figure 2d). Expression analysis further showed that SOCS2, EGR1, ATF3, KLF6, DPP4, and DPEP1 were downregulated in CKD, whereas HMOX1, IRF7, CD74, IRF1, IRF9, LCN2, S100A8, and CXCL10 were generally upregulated (Figure 2e).

### 3.2. Disease-Subtype Sensitivity Analyses Support Directionally Consistent SOCS2 Downregulation

Disease-subtype sensitivity analyses were performed to determine whether the pooled SOCS2 signal was driven by specific renal diagnoses. In GSE104954, median SOCS2 expression was lower than that of same-platform living-donor controls across all 10 renal disease categories. Because platform and disease composition were strongly confounded, inferential comparisons were restricted to within-platform analyses. On GPL22945, SOCS2 downregulation was particularly pronounced in ANCA-associated vasculitis (*n* = 21; Cohen’s d = −5.70; BH-adjusted *p* = 5.47 × 10^−7^) and diabetic nephropathy (*n* = 7; Cohen’s d = −4.39; BH-adjusted *p* = 3.88 × 10^−4^). All disease categories represented on GPL24120 also showed lower SOCS2 expression than the three same-platform controls; however, these comparisons were considered exploratory because of the very small control sample.

In GSE180394, SOCS2 expression was directionally lower in all five prespecified renal disease categories, with effect sizes of −0.71 for lupus nephritis, −1.00 for FSGS/FGGS, −0.81 for diabetic nephropathy, −0.99 for IgA nephropathy, and −1.18 for other renal diseases. Most subgroup comparisons did not remain statistically significant after multiple-testing correction, consistent with the limited subgroup sample sizes.

Donor-level PT-cell analysis in GSE183276 showed similarly reduced SOCS2 expression in DKD (*n* = 12; Cohen’s d = −3.68; BH-adjusted *p* = 1.07 × 10^−5^). Hypertensive CKD also showed a large negative effect size (*n* = 3; Cohen’s d = −3.38), although this subgroup was too small for reliable disease-specific inference. Collectively, these sensitivity analyses showed a consistent direction of SOCS2 downregulation across multiple renal disease categories, but they do not establish that the magnitude of SOCS2 reduction is equivalent across all CKD etiologies.

### 3.3. Identification of Ferro-Aging-Related Hub Genes in CKD Using Multiple Machine-Learning Algorithms

**Figure 3:**
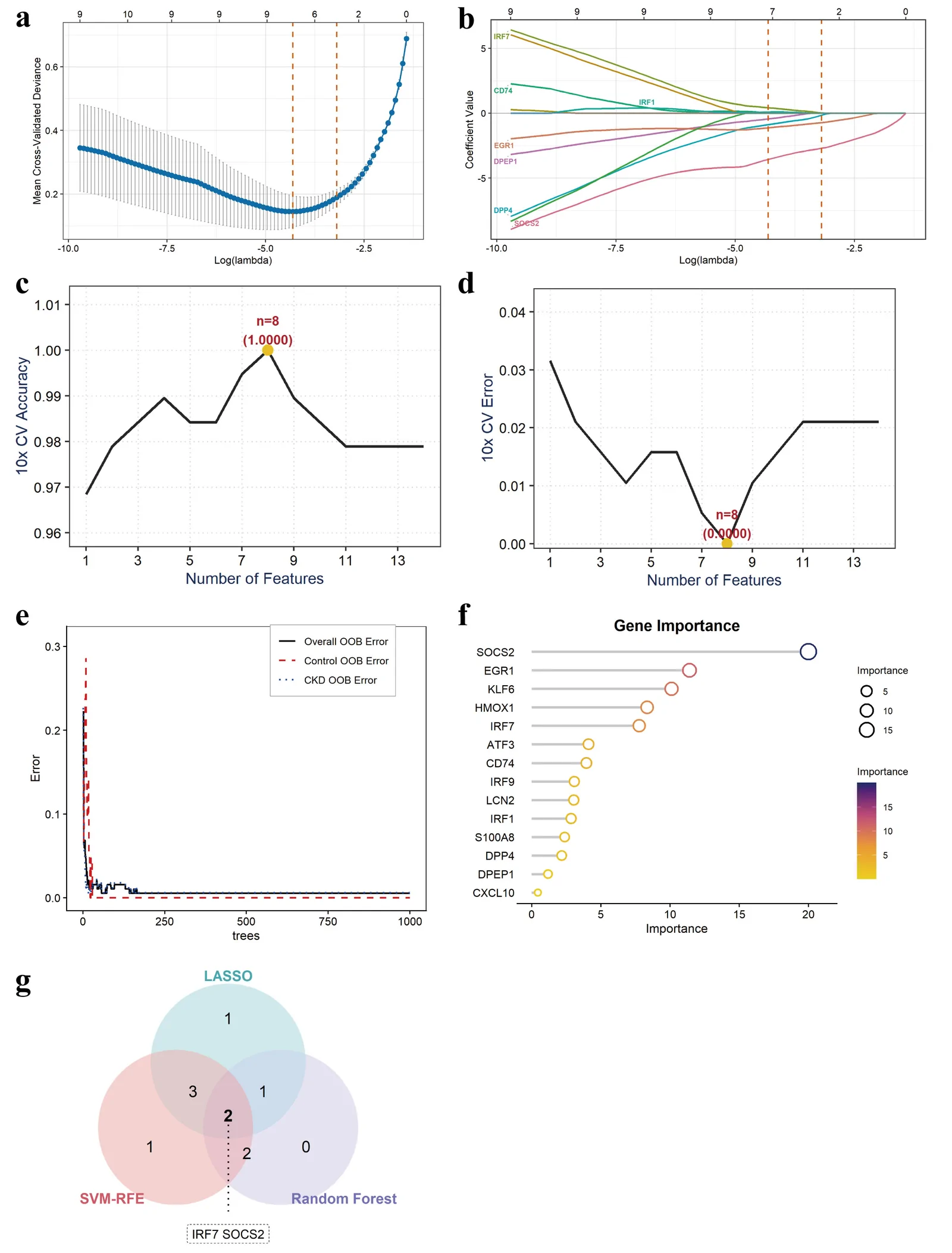
Machine-learning identification of ferro-aging-related candidate hub genes in CKD. (**a**,**b**) LASSO 10-fold cross-validation and coefficient-path plots; (**c**,**d**) SVM-RFE accuracy and error curves; (**e**,**f**) random-forest out-of-bag error and gene-importance ranking; (**g**) intersection of genes selected by the three algorithms.

The 14 ferro-aging-related genes identified by intersection analysis were used as candidate variables for feature selection with LASSO, SVM-RFE, and random forest. The LASSO cross-validation curve showed a gradual reduction in the number of model features as the penalty parameter increased, with an optimal parameter obtained near the minimum cross-validation error (Figure 3a). The coefficient-path plot further illustrated the progressive shrinkage of candidate-gene coefficients toward zero as λ increased; SOCS2, IRF7, and several other genes retained nonzero coefficients over a broad parameter range (Figure 3b). SVM-RFE achieved the highest 10-fold cross-validation accuracy and lowest cross-validation error when eight features were retained (Figure 3c,d). The random-forest out-of-bag error decreased rapidly and stabilized as the number of trees increased, indicating good internal model fit (Figure 3e). Variable-importance ranking identified SOCS2 as the most important feature, followed by EGR1, KLF6, HMOX1, IRF7, and other genes (Figure 3f). Intersection of the outputs from the three algorithms identified IRF7 and SOCS2 as the common hub genes (Figure 3g). Feature-selection stability was further examined across the 25 outer training sets of the repeated nested cross-validation. SOCS2 entered the ferro-aging candidate set, LASSO model, and random-forest selection in 100% of outer training sets, was retained by SVM-RFE in 92%, and was included in the three-method consensus in 92%. By comparison, IRF7 was included in the three-method consensus in 36% of outer training sets. Thus, although the full-cohort workflow recovered both IRF7 and SOCS2, SOCS2 showed substantially greater resampling stability (Supplementary Figure S2).

### 3.4. Exploratory Discriminatory Analysis of IRF7 and SOCS2 Across CKD Cohorts

**Figure 4:**
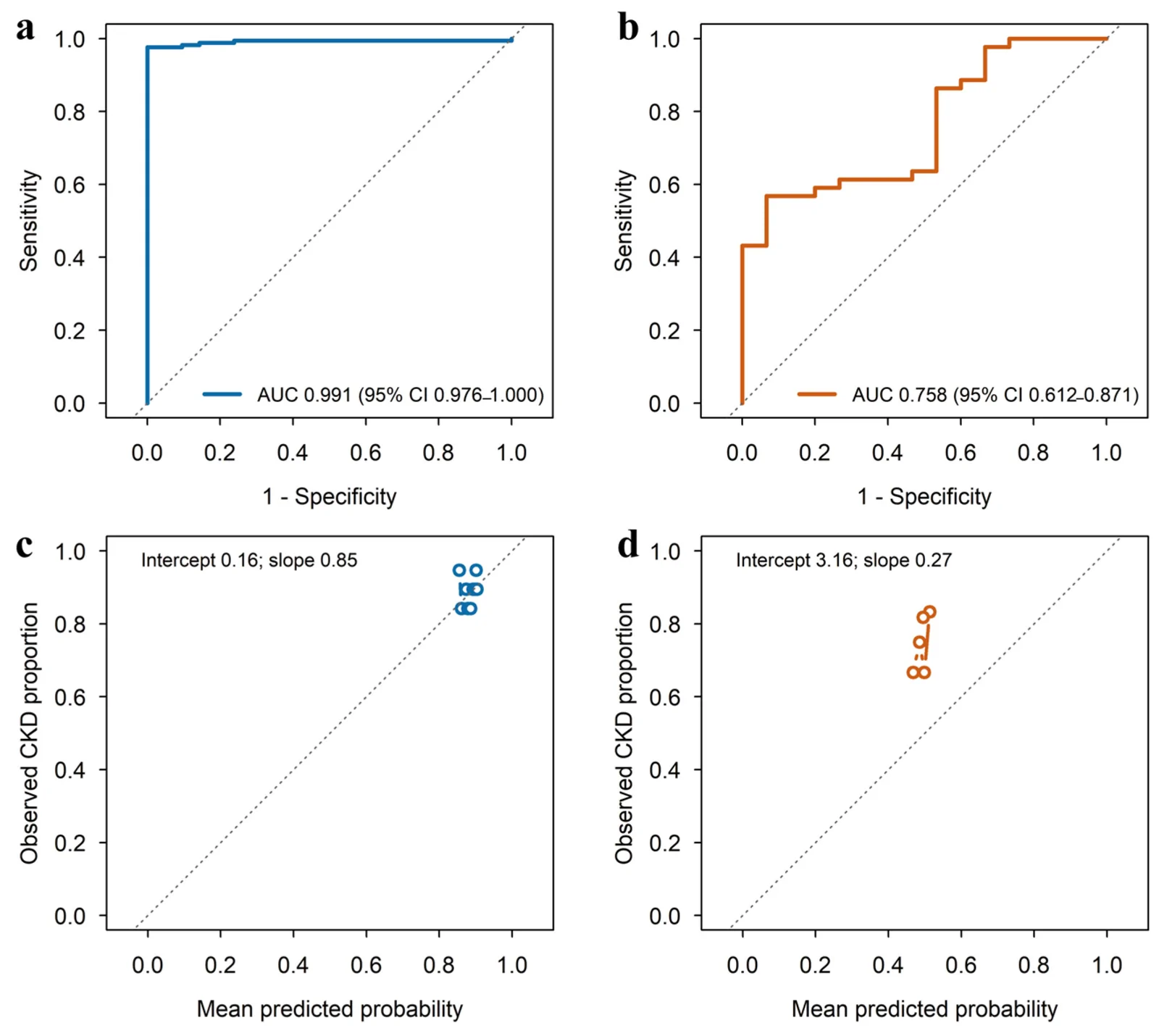
Exploratory discriminatory and calibration performance of the IRF7/SOCS2 tissue-expression classifier. (**a**) Receiver operating characteristic curve based on subject-level out-of-fold probabilities from fivefold nested cross-validation repeated five times in GSE104954. (**b**) Receiver operating characteristic curve in GSE180394 after applying the original GSE104954 equation without refitting. (**c**) Internal calibration based on deciles of the out-of-fold probabilities. (**d**) External calibration based on quintiles of the locked model probabilities. The GSE104954 cutoff of 0.899343941 was applied unchanged to GSE180394. Confidence intervals were obtained using 2000 stratified bootstrap resamples. No coefficients, preprocessing parameters, or thresholds were estimated from GSE180394 outcomes.

A multivariable logistic regression model based on IRF7 and SOCS2 was evaluated as an exploratory tissue-expression classifier rather than as a clinical diagnostic tool. When the complete development workflow was evaluated by fivefold nested cross-validation repeated five times, the subject-level out-of-fold AUC was 0.991 (95% CI, 0.976–1.000). Sensitivity, specificity, balanced accuracy, and accuracy were 0.964 (95% CI, 0.935–0.988), 0.905 (95% CI, 0.762–1.000), 0.935 (95% CI, 0.860–0.988), and 0.958 (95% CI, 0.926–0.984), respectively (Figure 4a; Supplementary Table S2). The out-of-fold Brier score was 0.020 (95% CI, 0.009–0.036), the calibration intercept was 0.155 (95% CI, −0.399–0.865), and the calibration slope was 0.851 (95% CI, 0.436–18.696) (Figure 4c; Supplementary Table S2). The wide confidence interval for the calibration slope indicates substantial uncertainty because the discovery cohort contained only 21 controls.

The full-cohort workflow recovered IRF7 and SOCS2 and reproduced the reported equation. Across the 25 outer training sets, SOCS2 was included in the three-method consensus in 92%, compared to 36% for IRF7, indicating substantially greater resampling stability for SOCS2 (Supplementary Figure S2).

In GSE180394, the unchanged training-derived model yielded an AUC of 0.758 (95% CI, 0.612–0.871) (Figure 4b). At the prespecified discovery-cohort cutoff of 0.899343941, sensitivity was 0.409 (95% CI, 0.273–0.545), specificity was 1.000 (95% CI, 1.000–1.000), balanced accuracy was 0.705 (95% CI, 0.636–0.773), and accuracy was 0.559 (95% CI, 0.458–0.661) (Supplementary Table S2). External calibration was limited, with a Brier score of 0.282 (95% CI, 0.192–0.371), a calibration intercept of 3.162 (95% CI, 1.849–4.625), and a calibration slope of 0.275 (95% CI, 0.150–0.453) (Figure 4d; Supplementary Table S2). No model coefficients or decision thresholds were re-estimated in the external cohort.

These findings indicate strong internal discrimination but only moderate external discrimination and poor external probability calibration, supporting interpretation of the model as exploratory rather than clinically validated. SOCS2 remained the more stable individual candidate across resampling and independent expression analyses. Accordingly, the model should be interpreted as describing cross-cohort tissue-expression discrimination and not as a clinically deployable CKD diagnostic test.

### 3.5. Single-Cell Analysis Identifies SOCS2-Associated Ferroptotic Changes in PT Cells

**Figure 5:**
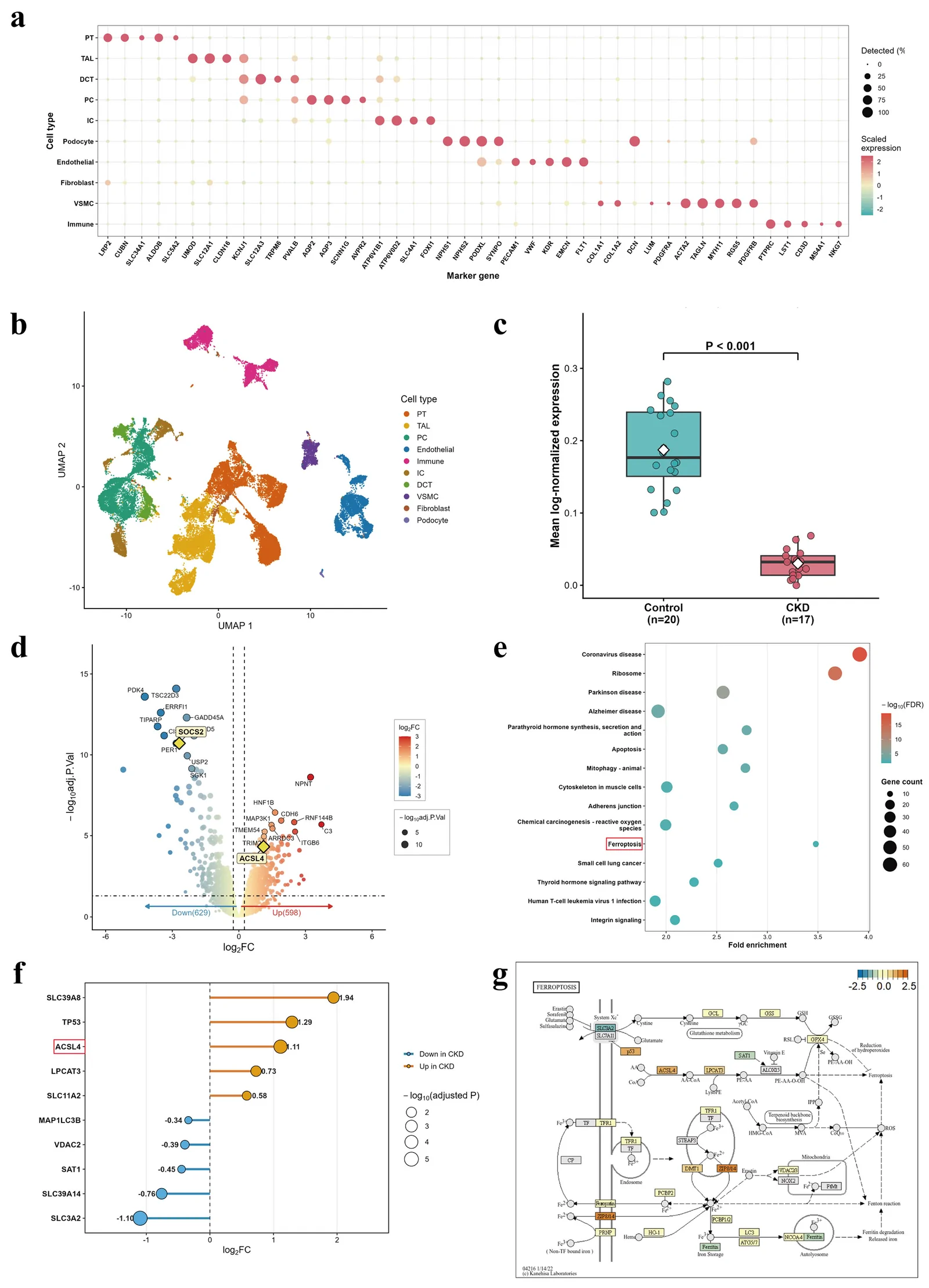
Single-cell characterization of SOCS2 expression and ferroptosis-related transcriptomic features in PT cells. (**a**) Bubble plot of marker genes; (**b**) UMAP plot of cell types in GSE183276; (**c**) donor-level SOCS2 expression in PT cells; each point represents one unique donor (18 controls and 15 CKD donors), and group differences were assessed using the Wilcoxon rank-sum test. Boxplots show the median and interquartile range, with whiskers extending to 1.5 × the interquartile range. (**d**) Volcano plot of donor-level pseudobulk differential expression between CKD and control PT cells; *p* values were adjusted using the Benjamini–Hochberg method. (**e**) KEGG enrichment analysis of PT-cell differentially expressed genes; (**f**,**g**) ferroptosis-related differentially expressed genes and their mapping to the KEGG ferroptosis pathway.

After quality control, integration, clustering, and cell-type annotation of the GSE183276 single-cell transcriptomic data, the major renal cell types identified included PT, TAL, DCT, principal cells, intercalated cells, podocytes, endothelial cells, fibroblasts, vascular smooth muscle cells, and immune cells (Figure 5b). Canonical marker genes exhibited relatively cell-type-specific expression patterns across cell populations, supporting the reliability of the annotations (Figure 5a). At the donor level, mean SOCS2 expression in PT cells was markedly lower in the CKD group than in the control group (0.0284 vs. 0.1902; Wilcoxon *p* = 1.16 × 10^−6^; BH-adjusted *p* = 1.16 × 10^−5^; Hedges’ g = −3.80; Figure 5c), indicating a large between-group difference. Pseudobulk differential expression analysis of PT cells identified 598 upregulated and 629 downregulated genes. SOCS2 was significantly downregulated, whereas ACSL4, which promotes polyunsaturated fatty-acid activation and lipid peroxidation, was significantly upregulated (Figure 5d). KEGG analysis further showed that the PT-cell differentially expressed genes were enriched in the ferroptosis pathway (Figure 5e). Among pathway-related genes, SLC39A8, TP53, ACSL4, LPCAT3, and SLC11A2 were upregulated in CKD, whereas MAP1LC3B, VDAC2, SAT1, SLC39A14, and SLC3A2 were downregulated (Figure 5f). These transcriptomic changes are consistent with alterations in pathways related to iron handling, antioxidant defense, and lipid-peroxidation susceptibility. However, because iron accumulation, lipid peroxidation, cellular senescence, and ferroptotic cell death were not directly measured, these findings should be interpreted as molecular features compatible with a ferro-aging-related state rather than evidence of an established ferro-aging phenotype in CKD PT cells.

### 3.6. In Silico SOCS2 Perturbation Predicts Remodeling of Mitochondrial and Metabolic Regulatory Networks in PT Cells

**Figure 6:**
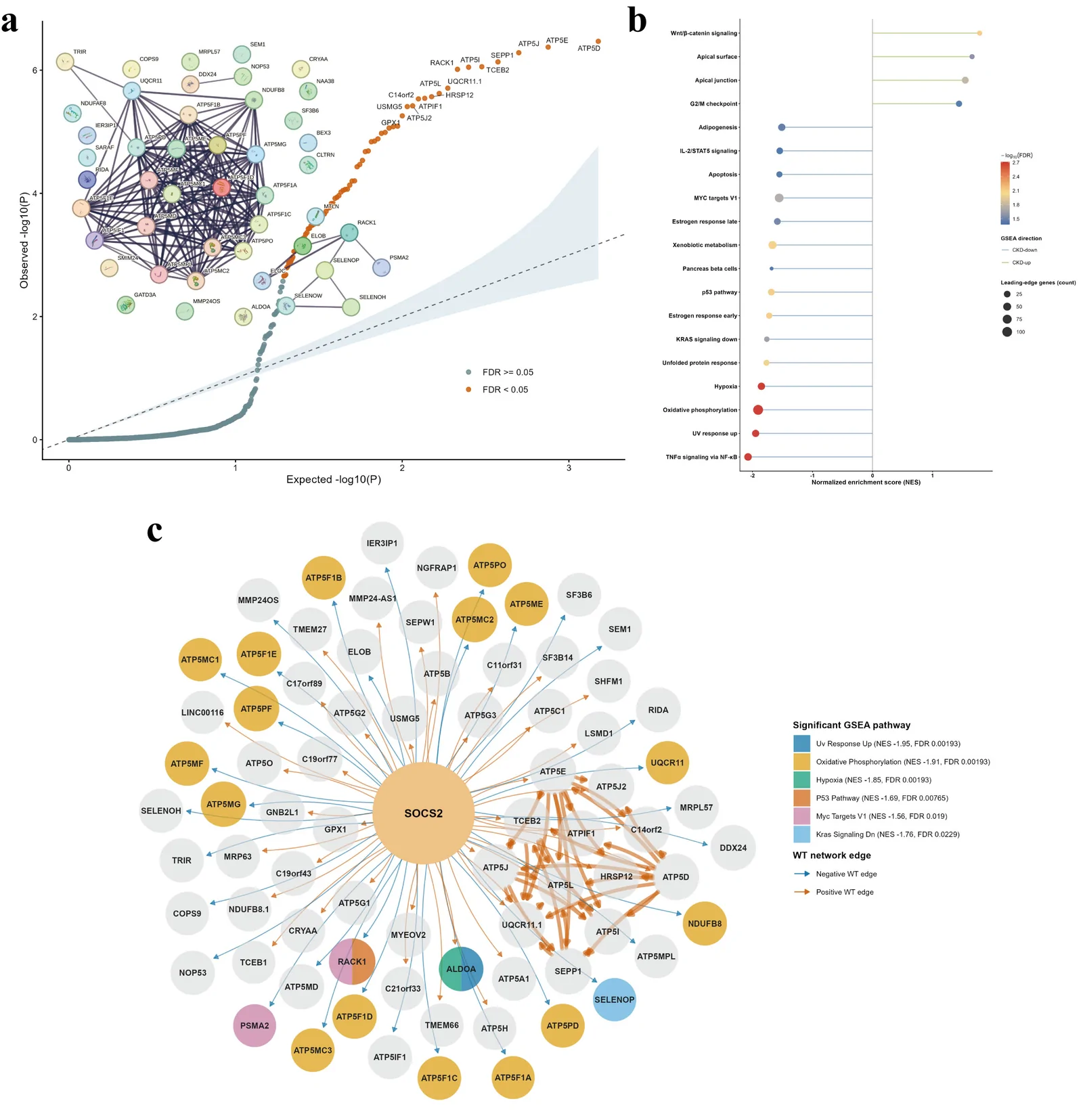
Regulatory network and GSEA associated with in silico SOCS2 network perturbation in control PT cells. (**a**) Quantile–quantile plot and network of significantly perturbed genes; significance was assessed using the χ^2^-based procedure implemented in scTenifoldKnk with Benjamini–Hochberg correction, and FDR < 0.05 was considered significant; (**b**) GSEA enrichment plots; (**c**) regulatory network following in silico SOCS2 network perturbation.

To investigate the predicted network-level consequences of SOCS2 perturbation in PT cells, SOCS2 was virtually deleted from the inferred PT-cell gene regulatory network using scTenifoldKnk. ATP5D, ATP5E, ATP5J, SEPP1, RACK1, and other genes exhibited significant regulatory perturbations, with ATP5-family members showing the most prominent changes (Figure 6a). At an FDR threshold of < 0.05, 77 significantly perturbed nontarget genes were identified, and an SOCS2-centered regulatory network containing 112 edges was constructed (Figure 6c). Mitochondria-related genes, including ATP5-family members, UQCR11.1, ATPIF1, and USMG5, formed a prominent module within the network, which also included oxidative stress and metabolic genes such as GPX1, SEPP1, and ALDOA. Hallmark GSEA identified 19 significantly enriched pathways (Figure 6b). In CKD PT cells, oxidative phosphorylation, hypoxia, and TNF-α signaling via NF-κB were negatively enriched, whereas Wnt/β-catenin signaling, apical junction, and the G2/M checkpoint were positively enriched. Oxidative phosphorylation showed the greatest overlap with the SOCS2-perturbed regulatory network, involving 16 genes. Collectively, virtual SOCS2 perturbation was associated with predicted remodeling of networks related to mitochondrial oxidative phosphorylation, oxidative stress, and metabolic homeostasis in PT cells. These network-level changes are compatible with molecular processes relevant to the proposed ferro-aging framework but do not establish a causal role for SOCS2 in ferro-aging or tubular injury.

## 4. Discussion

By integrating bulk transcriptomics, machine learning, single-cell transcriptomics, and in silico knockout analysis, this study identified IRF7 and SOCS2 from the intersection of CKD-associated differentially expressed genes and ferro-aging-related genes. Compared to IRF7, SOCS2 showed a more consistent downregulation pattern across the training cohort, external validation cohort, and single-cell dataset and demonstrated good discriminatory ability for CKD, suggesting that SOCS2 is a key candidate gene associated with tubular injury in CKD. SOCS2 is a negative regulator of cytokine signaling that limits the sustained activation of inflammatory and growth-factor pathways. Kidney-disease models have shown that increasing SOCS2 expression can alleviate inflammation, tissue injury, and fibrosis [27]. The persistent reduction in SOCS2 observed here may therefore indicate the impairment of endogenous inflammatory negative-feedback mechanisms in the kidney, rendering tubular cells more susceptible to chronic inflammation and metabolic stress.

IRF7, the other hub gene consistently selected by all three machine-learning algorithms, may also be relevant to CKD-related inflammatory and fibrotic responses. Recent evidence indicates that IRF7 can promote renal fibrosis by facilitating macrophage-to-myofibroblast transition through the IRF7–CTSS axis, supporting a role for sustained interferon-related signaling in chronic kidney injury [28]. IRF7 has also been implicated in iron-dependent lipid peroxidation and ferroptosis in nonrenal epithelial cells through the regulation of the miR-375-3p/SLC11A2 axis [29], suggesting a possible mechanistic link between IRF7-mediated inflammatory signaling and ferroptosis-related processes. However, IRF7 was not significantly differentially expressed in our external validation cohort, and direct evidence linking IRF7 to ferro-aging in CKD remains lacking; therefore, its relevance to ferro-aging should be considered preliminary.

Single-cell analysis further showed that SOCS2 downregulation occurred predominantly in PT cells. PT cells are responsible for glomerular-filtrate reabsorption, solute transport, and energy metabolism. Because of their high metabolic demand, they are particularly vulnerable to hypoxia, oxidative stress, and toxic metabolites. Persistent or recurrent PT-cell injury can lead to tubular atrophy and can also promote tubulointerstitial damage through the release of inflammatory and profibrotic signals [30]. Previous studies have shown that some injured PT cells fail to regain a mature transport and metabolic phenotype. Instead, they enter a failed-repair state characterized by inflammatory, senescent, and profibrotic features [31,32]. These observations are consistent with our finding that SOCS2 downregulation was concentrated in PT cells and suggest that reduced SOCS2 expression may be associated with persistent tubular injury.

PT cells have high energy demands, and their reabsorptive and transport functions depend largely on fatty-acid oxidation and mitochondrial oxidative phosphorylation. Impaired fatty-acid oxidation reduces ATP production and promotes lipid accumulation and oxidative stress. These changes can contribute to tubular atrophy and interstitial fibrosis [33]. Conversely, the restoration of oxidative metabolism or fatty-acid oxidation has been shown to improve mitochondrial homeostasis and attenuate tubular injury and renal fibrosis [34,35]. Together, these findings suggest that reduced SOCS2 may be associated not only with inflammatory signaling but also with altered mitochondrial bioenergetics and redox homeostasis in PT cells.

Ferroptosis-related tubular injury has likewise been reported in diabetic, fibrotic, and hypertension-associated CKD models, including ACSL4-dependent mechanisms [36,37,38]. However, these studies concern ferroptotic injury rather than ferro-aging and therefore provide contextual support rather than direct evidence for a ferro-aging phenotype in the present study.

An alternative explanation for the bulk-tissue SOCS2 signal is variation in tissue composition rather than a uniform cell-intrinsic change. CKD kidney tissue contains variable proportions of injured tubular cells, immune cells, fibroblasts, and other stromal populations, and these differences may influence bulk transcriptomic expression estimates. The donor-level single-cell analysis showed reduced SOCS2 expression within PT cells themselves, which argues against tissue-composition differences being the sole explanation for the bulk-tissue association. However, residual effects of cell-state composition, inflammatory-cell infiltration, and fibrosis cannot be excluded.

The CKD cohorts analyzed here were etiologically heterogeneous, and pooled CKD-versus-control comparisons could therefore potentially be driven by individual renal disease categories. Our platform-stratified sensitivity analyses showed that SOCS2 expression was directionally reduced across all evaluated disease categories in GSE104954 and across the five prespecified disease groups in GSE180394. Donor-level PT-cell analysis further demonstrated marked SOCS2 reduction in DKD and a similar direction in hypertensive CKD. These findings argue against the pooled association being attributable exclusively to a single renal diagnosis. However, subgroup sample sizes were limited, particularly on GPL24120, in GSE180394, and for hypertensive CKD in GSE183276. Therefore, the present data support cross-disease directional consistency rather than the equivalence of SOCS2 downregulation across all CKD etiologies.

Control selection and technical heterogeneity represent additional potential sources of variation across cohorts. GSE104954 used living-donor kidney tissue as the control source, whereas GSE180394 included both living-donor samples and non-neoplastic kidney tissue obtained from tumor nephrectomy. Although these tissues were classified as non-CKD controls in the source datasets, differences in tissue procurement and clinical context may influence baseline gene expression profiles. In addition, GSE104954 combined two microarray platforms with an imbalanced distribution of CKD and control samples. Platform-adjusted modeling and PCA reduced platform-associated variation, but statistical correction cannot fully eliminate confounding introduced by the original study design. These factors should therefore be considered when interpreting cross-cohort differences in SOCS2 expression and classifier performance.

This study has several limitations. First, the single-cell analyses are observational, and the scTenifoldKnk analysis represents a computational perturbation of an inferred regulatory network rather than an experimental SOCS2 knockout. Therefore, the predicted remodeling of mitochondrial oxidative phosphorylation, oxidative stress, and metabolic networks cannot establish that SOCS2 directly regulates these processes. The overlap between SOCS2-perturbed networks and CKD-associated transcriptional pathways should be interpreted as hypothesis-generating evidence that prioritizes candidate mechanisms for experimental testing. Functional gain- and loss-of-function studies in cell culture and animal models are required to determine whether SOCS2 directly alters mitochondrial respiration, ATP production, Fe^2+^ accumulation, lipid ROS, ACSL4, GPX4, or related ferro-aging-associated phenotypes. Second, although the complete model-development workflow was evaluated using repeated nested cross-validation, uncertainty remains because the discovery cohort included only 21 controls. The nested analysis confirmed high internal discrimination, but feature-selection stability differed substantially between SOCS2 and IRF7: SOCS2 was included in the three-method consensus in 92% of outer training sets, whereas IRF7 was included in 36%. More importantly, the unchanged two-gene model showed only moderate discrimination in GSE180394 (AUC, 0.758) and poor external calibration (calibration intercept, 3.162; calibration slope, 0.275) (Figure 4b,d). At the prespecified discovery-cohort cutoff of 0.899343941, sensitivity was only 0.409. These findings indicate that the model is not yet transportable as a calibrated clinical prediction tool and should be considered exploratory rather than clinically validated. Further validation and, if clinically justified, prespecified recalibration are required in larger, prospectively collected cohorts. Third, all transcriptomic datasets analyzed in this study were retrospectively obtained from the GEO database, and detailed information regarding ethnicity, population background, CKD stage, underlying renal disease, and other clinical characteristics was incomplete or unavailable for some cohorts. Consequently, we were unable to systematically evaluate ethnic or population-specific differences or fully adjust for potential clinical confounders that may influence gene expression patterns. These limitations may restrict the generalizability of the present findings. Future studies should therefore validate SOCS2 expression and its associated molecular features in larger, prospectively collected, clinically well-characterized, and ethnically diverse CKD cohorts.

In summary, SOCS2 is a hypothesis-generating candidate associated with a ferro-aging-related transcriptomic signature in CKD PT cells, including alterations in inflammatory negative-feedback signaling, mitochondrial metabolism, and oxidative stress-related networks. These findings do not establish ferro-aging or a causal role of SOCS2 and support further functional validation in cellular and preclinical models of CKD.

## Figures and Tables

**Table 1:** Characteristics of the transcriptomic cohorts included in the study.

Dataset	Platform and Tissue Compartment	Renal Disease Composition	Control Source	Inclusion and Exclusion	Available Clinical Information
GSE104954	GPL22945 and GPL24120; microdissected tubulointerstitial kidney biopsy tissue	SLE (*n* = 33), IgAN (*n* = 25), ANCA-associated vasculitis (*n* = 21), hypertensive nephropathy (*n* = 20), membranous nephropathy (*n* = 17), diabetic nephropathy (*n* = 17), FSGS (*n* = 13), MCD (*n* = 13), thin basement membrane disease (*n* = 6), mixed FSGS/MCD (*n* = 4)	21 living-donor kidney samples; 18 on GPL22945 and 3 on GPL24120	169 CKD samples and 21 living-donor controls were included; 5 tumor-nephrectomy samples were excluded	Renal diagnosis available; age, eGFR, CKD stage, treatment, and quantitative pathological severity unavailable
GSE180394	GPL19983; microdissected tubular compartment	Lupus nephritis (*n* = 14), FSGS/FGGS (*n* = 9), DN (*n* = 4), IgAN (*n* = 4), other renal diseases (*n* = 13)	9 living donors and 6 non-neoplastic kidney samples from tumor nephrectomy	All 59 samples included 44 renal disease samples and 15 controls	Diagnosis and control source available; age, eGFR, CKD stage, and quantitative fibrosis data unavailable
GSE183276	10× single-cell RNA sequencing; kidney tissue	DKD (12 donors), hypertensive CKD (3 donors)	18 healthy reference donors	33 CKD/control donors, 37 libraries, and 45,112 cells included; 12 AKI donors excluded	Sex available: control 11F/7M, CKD 10F/5M; age and eGFR unavailable; ethnicity unavailable for controls

## Data Availability

The datasets analyzed in this study are publicly available in the National Center for Biotechnology Information Gene Expression Omnibus database under accession numbers GSE104954, GSE180394 and GSE183276. Further information regarding data processing and analysis is available from the corresponding authors upon reasonable request.

## Supplementary Materials

The following supporting information can be downloaded at https://www.mdpi.com/article/10.3390/biomedicines14092047/s1.

## Abbreviations

The following abbreviations are used in this manuscript:

ACSL4	acyl-CoA synthetase long-chain family member 4
AKI	acute kidney injury
ATP	adenosine triphosphate
AUC	area under the receiver operating characteristic curve
BH	Benjamini–Hochberg
CKD	chronic kidney disease
DCT	distal convoluted tubule
DEG	differentially expressed gene
FDR	false discovery rate
GEO	Gene Expression Omnibus
GPX4	glutathione peroxidase 4
GSEA	gene set enrichment analysis
IC	intercalated cell
IRF7	interferon regulatory factor 7
LASSO	least absolute shrinkage and selection operator
MAD	median absolute deviation
MSigDB	Molecular Signatures Database
NAD	nicotinamide adenine dinucleotide
NES	normalized enrichment score
NF-κB	nuclear factor kappa B
PC	principal cell
PT	proximal tubular cell
ROC	receiver operating characteristic
ROS	reactive oxygen species
SNN	shared nearest-neighbor
SOCS2	suppressor of cytokine signaling 2
SVM-RFE	support vector machine–recursive feature elimination
TAL	thick ascending limb
TMM	trimmed mean of M-values
