# Supplementary material for: SOCS2 Downregulation Is Associated with a Ferro-Aging-Related Transcriptomic Signature in Proximal Tubular Cells in Chronic Kidney Disease
Source: Biomedicines. 2026 Sep 11;14(9):2047. doi: 10.3390/biomedicines14092047 (PMC13604860; doi:10.3390/biomedicines14092047)
Supplement: Supplementary file 1 [file biomedicines-14-02047-s001.zip › Supplementary Figures.pdf]

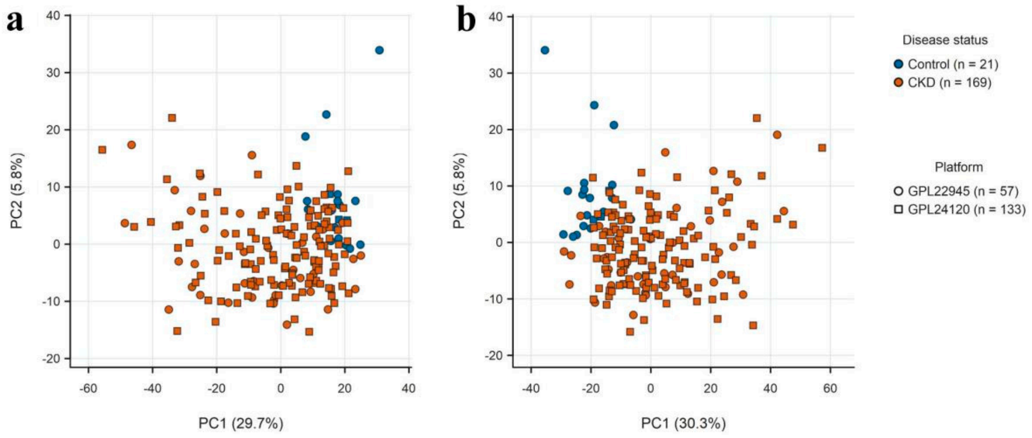

Supplementary Figure S1. Principal component analysis of GSE104954 before and after platform correction. PCA was performed using all 12,042 shared gene symbols across 190 samples, including 169 CKD and 21 control samples. (a) PCA of the merged expression matrix after probe-to-gene annotation, aggregation of multiple probes mapping to the same gene symbol, and quantile normalization, but before platform correction. (b) PCA after correction for platform effects using limma::removeBatchEffect, with the CKD–control design matrix retained during correction. Each point represents one sample; color indicates disease status, and shape indicates the microarray platform. The percentages shown on the axes represent the variance explained by the corresponding principal components.

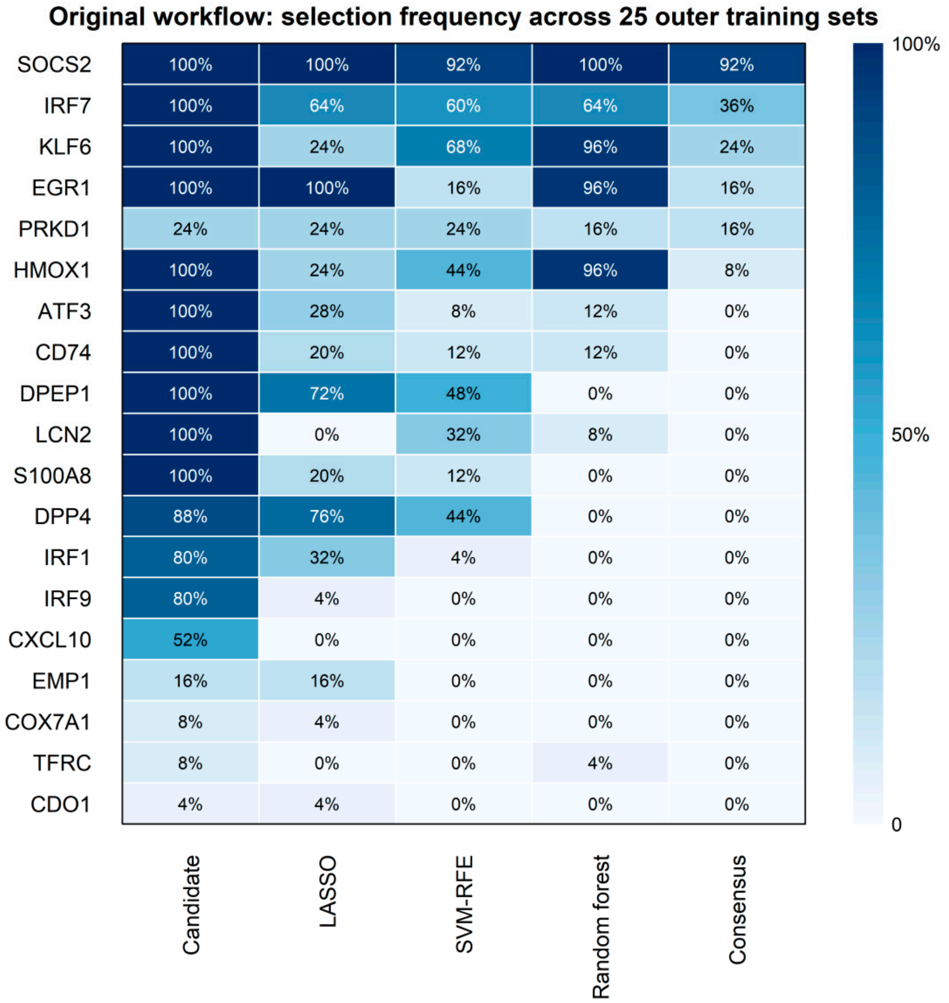

Supplementary Figure S2. Feature-selection stability across 25 outer training sets in the repeated nested cross-validation. Candidate denotes genes meeting the fold-specific differential-expression criteria and belonging to the prespecified ferro-aging gene set. Consensus denotes genes selected by LASSO, SVM-RFE, and random forest in the same outer training set. Values indicate the percentage of outer training sets in which each gene was retained.
